# Certain inequalities involving the k-Struve function

**DOI:** 10.1186/s13660-017-1343-x

**Published:** 2017-04-08

**Authors:** Kottakkaran Sooppy Nisar, Saiful Rahman Mondal, Junesang Choi

**Affiliations:** 1 Department of Mathematics, College of Arts and Science-Wadi Aldawaser, Prince Sattam bin Abdulaziz University, Wadi Ad-Dawasir, Riyadh region 11991 Saudi Arabia; 2 Department of Mathematics and Statistics, College of Science, King Faisal University, Hofuf, Al-Hasa 31982 Saudi Arabia; 3 Department of Mathematics, Dongguk University, Gyeongju, 38066 Republic of Korea

**Keywords:** 33C10, 26D07, k-Struve function, k-gamma function, k-beta function, k-digamma function, Turán-type inequalities

## Abstract

We aim to introduce a k-Struve function and investigate its various properties, including mainly certain inequalities associated with this function. One of the inequalities given here is pointed out to be related to the so-called classical Turán-type inequality. We also present a differential equation, several recurrence relations, and integral representations for this k-Struve function.

## Introduction and preliminaries

Díaz and Pariguan [1] introduced and investigated the so-called k-gamma function

1 $$ \Gamma_{\mathtt{k}}(x):= \int_{0}^{\infty}t^{x-1}e^{-\frac{t^{\mathtt {k}}}{\mathtt{k}}} \,dt\quad \bigl(\Re(x)>0; \mathtt{k} \in\mathbb{R}^{+} \bigr). $$

Here and in the following, let $\mathbb{C}$, $\mathbb{R}$, $\mathbb {R}^{+}$, $\mathbb{N}$, and $\mathbb{Z}^{-}$ be the sets of complex numbers, real numbers, positive real numbers, positive integers, and negative integers, respectively, and let $\mathbb{N}_{0}:=\mathbb{N} \cup \{0\}$. For various properties of the k-gamma function and its applications to generalize other related functions such as k-beta function and k-digamma function, we refer the interested reader, for example, to [1–3] and the references cited therein.

Nantomah and Prempeh [2] defined the k-digamma function $\Psi_{\mathtt{k}}:=\Gamma_{\mathtt{k}}'/\Gamma_{\mathtt{k}}$ whose series representation is given as follows:

2 $$ \Psi_{\mathtt{k}}(t):=\frac{\log\mathtt{k}-\gamma}{\mathtt{k}}-\frac{1}{t} + \sum_{n=1}^{\infty}\frac{t}{n\mathtt{k}(n\mathtt{k}+t)}\quad \bigl( \mathtt {k} \in\mathbb{R}^{+}; t \in\mathbb{C} \setminus\mathtt{k} \mathbb{Z}^{-} \bigr), $$

where *γ* is the Euler-Mascheroni constant (see, e.g., [4], Section 1.2). A calculation yields

3 $$ \Psi_{\mathtt{k}}'(t)=\sum _{n=0}^{\infty}\frac{1}{(n\mathtt{k}+t)^{2}} \quad\bigl(\mathtt{k}, t \in \mathbb{R}^{+} \bigr). $$

Clearly, $\Psi_{\mathtt{k}}(t)$ is increasing on $(0, \infty)$.

Turán [5] proved that the Legendre polynomials $P_{n}(x)$ satisfy the following determinant inequality:

4 $$ \left\vert \begin{matrix} P_{n}(x) & P_{n+1}(x) \\ P_{n+1}(x) & P_{n+2}(x) \end{matrix} \right\vert \leq 0\quad (-1 \le x \le1; n \in\mathbb{N}_{0} ), $$

where the equality occurs only when $x = \pm1$. Recently, many researchers have applied the above classical inequality (4) in various polynomials and functions such as ultraspherical polynomials, Laguerre polynomials, Hermite polynomials, Bessel functions of the first kind, modified Bessel functions, and polygamma functions. Karlin and Szegö [6] named such determinants as in (4) Turánians.

In this paper, we consider the following k-Struve function (cf. [7], p. 496, Entry 12.1.3):

5 $$ \mathtt{S}_{\nu,c}^{\mathtt{k}}(x):=\sum _{r=0}^{\infty}\frac {(-c)^{r}}{\Gamma_{\mathtt{k}}(r\mathtt{k}+\nu+\frac{3\mathtt {k}}{2})\Gamma(r+\frac{3}{2})} \biggl(\frac{x}{2} \biggr)^{2r+\frac{\nu}{\mathtt{k}}+1}. $$

Then we investigate the k-Struve function (5) as follows: We establish certain inequalities involving $\mathtt{S}_{\nu ,c}^{\mathtt{k}}$, one of which is shown to be related to the Turán-type inequality; we show that the k-Struve function satisfies a second-order non-homogeneous differential equation; and we present an integral representation and recurrence relations for the k-Struve function.

## Inequalities

The modified k-Struve function is given as

6 $$ L_{\nu}^{\mathtt{k}}(x):= \mathtt{S}_{\nu,-1}^{\mathtt{k}}(x), $$

which is normalized and denoted by $\mathcal{L}_{\nu}^{\mathtt{k}}$ as follows:

7 $$ \mathcal{L}_{\nu}^{\mathtt{k}}(x)= \biggl( \frac{2}{x} \biggr)^{\frac{\nu }{\mathtt{k}}} \Gamma_{\mathtt{k}} \biggl(\nu+ \frac{3\mathtt{k}}{2} \biggr)L_{\nu }^{\mathtt{k}}(x) =\sum _{r=0}^{\infty}f_{r}(\nu,\mathtt{k} ) x^{2r+1}, $$

where

$$\begin{aligned} f_{r}(\nu,\mathtt{k} ): =\frac{\Gamma_{\mathtt{k}} (\nu+\frac {3\mathtt{k}}{2} )}{ \Gamma_{\mathtt{k}}(r\mathtt{k}+\nu+\frac{3 \mathtt{k}}{2})\Gamma (r+\frac{3}{2}) 2^{2r+1}}. \end{aligned}$$

Here, we investigate monotonicity and log-convexity involving $\mathcal {L}_{\nu}^{\mathtt{k}}$. To do this, we recall some known useful properties which are given in the following lemma (see [8]).

### Lemma 1

*Consider the power series* $f(x)=\sum_{\mathtt{k}=0}^{\infty}a_{\mathtt{k}} x^{\mathtt{k}}$ *and* $g(x)=\sum_{\mathtt{k}=0}^{\infty}b_{\mathtt{k}} x^{\mathtt{k}}$, *where* $a_{\mathtt{k}} \in\mathbb{R}$ *and* $b_{\mathtt{k}} \in\mathbb{R}^{+} $ $(\mathtt{k} \in\mathbb{N}_{0})$. *Further suppose that both series converge on* $\vert x \vert < r$. *If the sequence* $\{a_{\mathtt{k}}/b_{\mathtt{k}}\}_{\mathtt{k}\geq0}$ *is increasing* (*or decreasing*), *then the function* $x \mapsto f(x)/g(x)$ *is also increasing* (*or decreasing*) *on* $(0,r)$.

*If both* *f* *and* *g* *are even*, *or both are odd functions*, *then the above results will be applicable*.

### Theorem 1

*Let* $\mathtt{k} \in\mathbb{R}^{+}$ *be fixed*. *Then the following statements hold*.

(i) *For* $\nu\geq\mu>-{3 \mathtt{k}}/{2}$, *then the function* $x \mapsto\mathcal{{L}}_{\mu}^{\mathtt{k}}(x)/\mathcal{L}_{\nu}^{\mathtt {k}}(x)$ *is increasing on* $\mathbb{R}$.
(ii) *The function* $\nu\mapsto\mathcal{L}_{\nu}^{\mathtt {k}}(x)$ *is decreasing for fixed* $x \in[0, \infty)$ *and increasing for fixed* $x \in(-\infty,0)$. *Also*, *the function* $\nu \mapsto\mathcal{L}_{\nu}^{\mathtt{k}}(x)$ *is log*-*convex on* $(-{3 \mathtt{k}}/{2}, \infty)$ *for fixed* $x \in\mathbb {R}^{+}$.
(iii) *The function* $\nu\mapsto L_{\nu+\mathtt{k}}^{\mathtt {k}}(x)/L_{\nu}^{\mathtt{k}}(x)$ *is decreasing on* $(-{3 \mathtt {k}}/{2}, \infty)$ *for fixed* $x \in\mathbb{R}^{+}$.

### Proof

To prove (i), recall the series in (7). Clearly,

$$\begin{aligned} \frac{\mathcal{L}_{\nu}^{\mathtt{k}}(x)}{\mathcal{L}_{\mu}^{\mathtt {k}}(x)} =\frac{\sum_{r=0}^{\infty}f_{r}(\nu, \mathtt{k}) x^{2r+1}}{\sum_{r=0}^{\infty}f_{r}(\mu,\mathtt{k}) x^{2r+1}}. \end{aligned}$$

Denote $w_{r}:={f_{r}(\nu, \mathtt{k})}/{f_{r}(\mu, \mathtt{k})}$. Then

$$\begin{aligned} w_{r}=\frac{\Gamma_{\mathtt{k}}(\nu+\frac{3}{2}\mathtt{k})\Gamma _{\mathtt{k}}(r\mathtt{k}+\mu+\frac{3}{2}\mathtt{k})}{ \Gamma_{\mathtt{k}}(r\mathtt{k}+\nu+\frac{3}{2}\mathtt{k})\Gamma _{\mathtt{k}}(\mu+\frac{3}{2}\mathtt{k})}. \end{aligned}$$

Appealing to relation (15), we find

$$\begin{aligned} \frac{w_{r+1}}{w_{r}}&=\frac{\Gamma_{\mathtt{k}}(\nu+\frac{3}{2}\mathtt {k})\Gamma_{\mathtt{k}}(r\mathtt{k}+\mathtt{k}+\mu+\frac{3}{2}\mathtt {k})}{\Gamma_{\mathtt{k}}(r\mathtt{k}+\mathtt{k}+\nu+\frac{3}{2}\mathtt {k})\Gamma_{\mathtt{k}}(\mu+\frac{3}{2}\mathtt{k})}\frac{\Gamma_{\mathtt {k}}(\mu+\frac{3}{2}\mathtt{k})\Gamma_{\mathtt{k}}(r\mathtt{k}+\nu+\frac {3}{2}\mathtt{k})}{\Gamma_{\mathtt{k}} (r\mathtt{k}+\mu+\frac{3}{2}\mathtt{k})\Gamma_{\mathtt{k}}(\nu+\frac {3}{2}\mathtt{k})} \\ &=\frac{(r\mathtt{k}+\mu+\frac{3}{2}\mathtt{k})\Gamma(r\mathtt{k}+\mu +\frac{3}{2}\mathtt{k})\Gamma_{\mathtt{k}}(r\mathtt{k}+\nu+\frac {3}{2}\mathtt{k})}{(r\mathtt{k}+\nu+\frac{3}{2}\mathtt{k})\Gamma (r\mathtt{k}+\nu+\frac{3}{2}\mathtt{k})\Gamma_{\mathtt{k}}(r\mathtt {k}+\mu+\frac{3}{2}\mathtt{k})} =\frac{r\mathtt{k}+\mu+\frac{3}{2}\mathtt{k}}{r\mathtt{k}+\nu+\frac {3}{2}\mathtt{k}}\leq1, \end{aligned}$$

whose last inequality is valid from the condition $\nu\geq\mu >-{3}\mathtt{k}/2$. Finally, the result (i) follows from Lemma 1.

For (ii), since $2\nu>-3\mathtt{k}$, we first observe the coefficients $f_{r}(\nu, \mathtt{k})>0$ for all $r \in\mathbb{N}_{0}$. Then the logarithmic derivative of $f_{r}(\nu, \mathtt{k})$ with respect to *ν* is

$$ \frac{f_{r}^{'}(\nu, \mathtt{k})}{f_{r}(\nu, \mathtt{k})}=\Psi_{\mathtt {k}} \biggl(\nu+\frac{3}{2}\mathtt{k} \biggr)-\Psi_{\mathtt{k}} \biggl(r\mathtt {k}+\nu+\frac{3}{2}\mathtt{k} \biggr)\leq0, $$

whose last inequality follows from (2). Since $f_{r}(\nu, \mathtt{k})>0$ $(r \in\mathbb{N}_{0}; 2\nu >-3\mathtt{k} )$, ${f_{r}}'(\nu, \mathtt{k})\leq0$ $(r \in \mathbb{N}_{0}; 2\nu>-3\mathtt{k} )$. Hence $\nu\mapsto f_{r}(\nu, \mathtt{k})$ is decreasing on $(-3 \mathtt {k}/2,\infty)$. This implies that, for $\mu\geq\nu>-{3}\mathtt{k}/{2}$,

$$ \sum_{r=0}^{\infty}f_{r}(\nu, \mathtt{k})x^{2r+1}\geq\sum_{r=0}^{\infty}f_{r}( \mu, \mathtt{k})x^{2r+1}\quad \bigl(x \in[0,\infty)\bigr) $$

and

$$ \sum_{r=0}^{\infty}f_{r}(\nu, \mathtt{k})x^{2r+1}\leq\sum_{r=0}^{\infty}f_{r}( \mu, \mathtt{k})x^{2r+1} \quad\bigl(x \in(-\infty, 0)\bigr). $$

This proves the first statement of (ii).

In view of (3), we have

$$ \frac{\partial^{2}}{\partial\nu^{2}} \bigl(\log\bigl(f_{r}(\nu, \mathtt {k})\bigr) \bigr) = \sum_{n=0}^{\infty} \biggl\{ \frac{1}{(n\mathtt{k}+\nu+\frac {3}{2}\mathtt{k})^{2}} -\frac{1}{(n\mathtt{k}+r\mathtt{k}+\nu+\frac{3}{2}\mathtt {k})^{2}} \biggr\} \geq0 $$

for all $\mathtt{k} \in\mathbb{R}^{+}$ and $2\nu>-3\mathtt{k}$. Therefore $\nu\mapsto f_{r}(\nu)$ is log-convex on $(-3\mathtt{k}/2 ,\infty)$. Since a sum of log-convex functions is log-convex, the second statement of (ii) is proved.

For (iii), it is obvious from (i) that

8 $$ \frac{d}{dx} \biggl(\frac{\mathcal{L}_{\mu}^{\mathtt{k}}(x)}{ \mathcal{L}_{\nu}^{\mathtt{k}}(x)} \biggr)\geq0, $$

for all $x \in\mathbb{R}^{+}$ and $\nu\geq\mu>-{3}\mathtt{k}/2$. In view of relation (7), (8) is equivalent to

9 $$\begin{aligned} \bigl(x^{-\frac{\mu}{\mathtt{k}}}\mathtt{L}_{\mu}^{\mathtt{k}}(x) \bigr)' \bigl(x^{-\frac{\nu}{\mathtt{k}}}\mathtt{L}_{\nu}^{\mathtt{k}}(x) \bigr)- \bigl(x^{-\frac{\mu}{\mathtt{k}}}\mathtt{L}_{\mu}^{\mathtt{k}}(x) \bigr) \bigl(x^{-\frac{\nu}{\mathtt{k}}}\mathtt{L}_{\nu}^{\mathtt{k}}(x) \bigr)'\geq0 \end{aligned}$$

for all $x \in\mathbb{R}^{+}$ and $\nu\geq\mu>-{3}\mathtt{k}/2$.

Considering (6) and setting $c=-1$ in (17) gives

10 $$ \frac{d}{dx} \bigl(x^{-\frac{\nu}{\mathtt{k}}}\mathtt{L}_{\nu}^{\mathtt {k}}(x) \bigr) =\frac{{2}^{-\frac{\nu}{\mathtt{k}}}}{ \sqrt{\pi} \Gamma_{\mathtt{k}} (\nu+\frac{\mathtt{3 k}}{2})} + x^{-\frac{\nu}{\mathtt{k}}}\mathtt{L}_{\nu+\mathtt{k}}^{\mathtt{k}}(x). $$

Applying (10) to inequality (9) and using (7), we obtain

11 $$\begin{aligned} & x^{-\frac{\mu+\nu}{\mathtt{k}}} \bigl\{ \mathtt{L}_{\mu+\mathtt {k}}^{\mathtt{k}}(x) \mathtt{L}_{\nu}^{\mathtt{k}}(x)-\mathtt{L}_{\nu+\mathtt{k}}^{\mathtt{k}}(x) \mathtt{L}_{\mu}^{\mathtt{k}}(x) \bigr\} \\ &\quad\geq \frac{{2}^{-\frac{\nu}{\mathtt{k}}}}{ \sqrt{\pi} \Gamma_{\mathtt{k}} (\nu+\frac{\mathtt{3 k}}{2})} x^{-\frac{\mu}{\mathtt{k}}}\mathtt{L}_{\mu }^{\mathtt{k}}(x) - \frac{{2}^{-\frac{\mu}{\mathtt{k}}}}{ \sqrt{\pi} \Gamma_{\mathtt{k}} (\mu+\frac{\mathtt{3 k}}{2})} x^{-\frac{\nu}{\mathtt{k}}}\mathtt{L}_{\nu }^{\mathtt{k}}(x) \\ &\quad = \frac{{2}^{-\frac{\mu+\nu}{\mathtt{k}}}}{ \sqrt{\pi} \Gamma_{\mathtt{k}} (\mu+\frac{\mathtt{3 k}}{2})\Gamma_{\mathtt{k}} (\nu+\frac{\mathtt{3 k}}{2})} \bigl(\mathcal{L}_{\mu}^{\mathtt{k}}(x) - \mathcal{L}_{\nu}^{\mathtt{k}}(x) \bigr) \geq0 \end{aligned}$$

for all $x \in\mathbb{R}^{+}$ and $\nu\geq\mu>-{3}\mathtt{k}/2$. Here, the last inequality in (11) follows from the first statement of (ii). Also, we find from (11) that

$$ \frac{L_{\mu+\mathtt{k}}^{\mathtt{k}}(x)}{L_{\mu}^{\mathtt{k}}(x)} - \frac{L_{\nu+\mathtt{k}}^{\mathtt{k}}(x)}{L_{\nu}^{\mathtt{k}}(x)}\geq0 $$

for all $x \in\mathbb{R}^{+}$ and $\nu\geq\mu>-{3}\mathtt{k}/2$. This proves (iii). □

### Remark 1

One of the most significant consequences of Theorem 1 is the Turán-type inequality for the function $\mathcal{L}_{\nu}^{\mathtt{k}}$. The log-convexity of $\mathcal{L}_{\nu}^{\mathtt{k}}$ (the last statement of (ii) in Theorem 1) implies

12 $$\begin{aligned} &\mathcal{L}_{\alpha\nu_{1}+(1-\alpha)\nu_{2}}^{\mathtt{k}}(x) \leq \bigl( \mathcal{L}_{\nu_{1}}^{\mathtt{k}} \bigr)^{\alpha}(x) \bigl( \mathcal{L}_{\nu_{2}}^{\mathtt{k}} \bigr)^{1-\alpha}(x) \\ &\quad\bigl(\alpha\in[0,1]; x, \mathtt{k} \in\mathbb{R}^{+}; \nu_{1}, \nu_{2} \in(-3\mathtt{k}/2, \infty) \bigr). \end{aligned}$$

Choosing $\alpha=1/2$ and setting $\nu_{1}=\nu-\mathtt{a}$ and $\nu_{2}=\nu +\mathtt{a}$ for some $\mathtt{a} \in\mathbb{R}$ in (12) yields the following reversed Turán-type inequality (cf. (4)):

13 $$\begin{aligned} &\bigl(\mathcal{L}_{\nu}^{\mathtt{k}}(x) \bigr)^{2} - \mathcal{L}_{\nu -\mathtt{a}}^{\mathtt{k}}(x) \mathcal{L}_{\nu+\mathtt{a}}^{\mathtt{k}}(x) \leq0 \\ &\quad\bigl(x, \mathtt{k} \in\mathbb{R}^{+}; \mathtt{a} \in\mathbb{R}, \nu \in \bigl( \vert \mathtt{a} \vert - 3 \mathtt{k}/2, \infty \bigr) \bigr). \end{aligned}$$

## Formulae for the k-Struve function

Here, we present a differential equation and recurrence relations regarding the k-Struve function $\mathtt{S}_{\nu, c}^{\mathtt{k}}$ (5).

### Proposition 1

*Let* $\mathtt{k} \in\mathbb{R}^{+}$ *and* $2\nu>-3 \mathtt{k}$. *Then the* k-*Struve function* $\mathtt{S}_{\nu, c}^{\mathtt{k}}$ (5) *satisfies the following second*-*order non*-*homogeneous differential equation*:

14 $$ x^{2} \frac{d^{2}y}{dx^{2}}+ x \frac{dy}{dx}+ \frac{1}{\mathtt{k}^{2}} \bigl(c \mathtt{k} x^{2}- \nu^{2} \bigr)y= \frac{4 (\frac{x}{2} )^{\frac{\nu}{\mathtt {k}}+1}}{ \mathtt{k} \Gamma_{\mathtt{k}}( \nu+ \frac{ \mathtt{k}}{2}) \Gamma( \frac{1}{2})}. $$

### Proof

By using the k-Struve function $\mathtt{S}_{\nu, c}^{\mathtt {k}}$ (5) and the functional relation

15 $$ \Gamma_{\mathtt{k}} (x + \mathtt{k})=x \Gamma_{\mathtt{k}} (x), $$

we find

$$\begin{aligned} &x^{2} \frac{d^{2}}{dx^{2}}\mathtt{S}_{\nu, c}^{\mathtt{k}} (x)+ x \frac {d}{dx}\mathtt{S}_{\nu, c}^{\mathtt{k}} (x) \\ &\quad=\sum_{r=0}^{\infty}\frac {(-c)^{r} (2r+\frac{\nu}{\mathtt{k}}+1 )^{2} }{ \Gamma_{\mathtt{k}}(r \mathtt{k}+ \nu+ \frac{3 \mathtt{k}}{2}) \Gamma(r +\frac{3}{2})} \biggl( \frac{x}{2} \biggr)^{2r+\frac{\nu}{\mathtt{k}}+1} \\ &\quad= \sum_{r=0}^{\infty}\frac{(-c)^{r} (2r+1) (2r+\frac{2\nu}{\mathtt {k}}+1 ) }{ \Gamma_{\mathtt{k}}(r \mathtt{k}+ \nu+ \frac{3 \mathtt {k}}{2}) \Gamma(r +\frac{3}{2})} \biggl(\frac{x}{2} \biggr)^{2r+\frac{\nu }{\mathtt{k}}+1}+ \frac{\nu^{2}}{\mathtt{k}^{2}} \mathtt{S}_{\nu, c}^{\mathtt{k}} (x) \\ &\quad=\frac{4}{\mathtt{k}} \sum_{r=0}^{\infty}\frac{(-c)^{r} (r+\frac {1}{2} ) (r \mathtt{k}+{\nu}+\frac{\mathtt{k}}{2} ) }{ \Gamma_{\mathtt{k}}(r \mathtt{k}+ \nu+ \frac{3 \mathtt{k}}{2}) \Gamma(r +\frac{3}{2})} \biggl(\frac{x}{2} \biggr)^{2r+\frac{\nu}{\mathtt{k}}+1}+ \frac{\nu^{2}}{\mathtt{k}^{2}}\mathtt{S}_{\nu, c}^{\mathtt{k}} (x) \\ &\quad=\frac{4}{\mathtt{k}}\frac{ (\frac{x}{2} )^{\frac{\nu}{\mathtt {k}}+1}}{ \Gamma_{\mathtt{k}}( \nu+ \frac{ \mathtt{k}}{2}) \Gamma( \frac {1}{2})} +\frac{4}{\mathtt{k}} \sum _{r=1}^{\infty}\frac{(-c)^{r} }{ \Gamma _{\mathtt{k}}(r \mathtt{k}+ \nu+ \frac{ \mathtt{k}}{2}) \Gamma(r +\frac {1}{2})} \biggl(\frac{x}{2} \biggr)^{2r+\frac{\nu}{\mathtt{k}}+1}+ \frac{\nu^{2}}{\mathtt{k}^{2}}\mathtt{S}_{\nu, c}^{\mathtt{k}} (x) \\ &\quad=\frac{4}{\mathtt{k}}\frac{ (\frac{x}{2} )^{\frac{\nu }{\mathtt{k}}+1}}{ \Gamma_{\mathtt{k}}( \nu+ \frac{ \mathtt{k}}{2}) \Gamma ( \frac{1}{2})}- \frac{c x^{2}}{\mathtt{k}}\mathtt{S}_{\nu, c}^{\mathtt{k}} (x) + \frac{\nu^{2}}{\mathtt{k}^{2}}\mathtt{S}_{\nu, c}^{\mathtt{k}} (x). \end{aligned}$$

This shows that $y=\mathtt{S}_{\nu, c}^{\mathtt{k}}(x)$ the differential equation (14). □

### Theorem 2

*Let* $\mathtt{k} \in\mathbb{R}^{+}$ *and* $2\nu>-3 \mathtt{k}$. *Then the following recurrence relations hold true*:

16 $$\begin{aligned} &\frac{d}{dx} \bigl(x^{\frac{\nu}{\mathtt{k}}}\mathtt{S}_{\nu,c}^{\mathtt {k}}(x) \bigr) =\frac{1}{\mathtt{k}} x^{\frac{\nu}{\mathtt{k}}}\mathtt{S}_{{\nu -\mathtt{k}}}^{\mathtt{k}}(x); \end{aligned}$$

17 $$\begin{aligned} &\frac{d}{dx} \bigl(x^{-\frac{\nu}{\mathtt{k}}}\mathtt{S}_{\nu,c}^{\mathtt {k}}(x) \bigr) =\frac{{2}^{-\frac{\nu}{\mathtt{k}}}}{ \sqrt{\pi} \Gamma_{\mathtt{k}} (\nu+\frac{\mathtt{3 k}}{2})} -c x^{-\frac{\nu}{\mathtt{k}}}\mathtt{S}_{\nu+\mathtt{k}, c}^{\mathtt {k}}(x); \end{aligned}$$

18 $$\begin{aligned} &\frac{1}{\mathtt{k}} \mathtt{S}_{{\nu-\mathtt{k}}}^{\mathtt{k}}(x)- c \mathtt{S}_{\nu+\mathtt{k}, c}^{\mathtt{k}}(x)= 2\frac{d}{dx} \mathtt{S}_{\nu,c}^{\mathtt{k}}(x)-\frac{ {(x/2)}^{\frac {\nu}{\mathtt{k}}}}{ \sqrt{\pi} \Gamma_{\mathtt{k}} (\nu+\frac{\mathtt{3 k}}{2})}; \end{aligned}$$

19 $$\begin{aligned} &\frac{1}{\mathtt{k}} \mathtt{S}_{{\nu-\mathtt{k}}}^{\mathtt{k}}(x)+ c \mathtt{S}_{\nu+\mathtt{k}, c}^{\mathtt{k}}(x)= \frac{2\nu}{x \mathtt{k} } \mathtt{S}_{\nu,c}^{\mathtt{k}}(x)+\frac{ {(x/2)}^{\frac{\nu}{\mathtt{k}}}}{ \sqrt{\pi} \Gamma_{\mathtt{k}} (\nu+\frac{\mathtt{3 k}}{2})}. \end{aligned}$$

### Proof

From (14) we have

$$x^{\frac{\nu}{\mathtt{k}}}\mathtt{S}_{\nu,c}^{\mathtt{k}}(x)= \sum _{r=0}^{\infty}\frac{(-c)^{r}}{ \Gamma_{\mathtt{k}}(r\mathtt{k}+\nu+\frac{3\mathtt{k}}{2})\Gamma (r+\frac{3}{2})} \frac{x^{2r+\frac{2\nu}{\mathtt{k}}+1}}{2^{2r+\frac{\nu}{\mathtt{k}}+1}}, $$

which, upon differentiating with respect to *x* and using relation (15), yields

$$\begin{aligned} \frac{d}{dx} \bigl(x^{\frac{\nu}{\mathtt{k}}}\mathtt{S}_{\nu ,c}^{\mathtt{k}}(x) \bigr) &=\sum_{r=0}^{\infty}\frac{(-c)^{r}(2r+\frac{2\nu}{\mathtt{k}}+1)}{ \Gamma_{\mathtt{k}}(r\mathtt{k}+\nu+\frac{3\mathtt{k}}{2})\Gamma (r+\frac{3}{2})} \frac{x^{2r+\frac{2\nu}{\mathtt{k}}}}{2^{2r+\frac{\nu}{\mathtt{k}}+1}} \\ & =\frac{x^{\frac{\nu}{\mathtt{k}}}}{\mathtt{k}}\sum_{r=0}^{\infty} \frac{(-c)^{r}}{ \Gamma_{\mathtt{k}} (r\mathtt{k}+\nu+\frac{\mathtt{k}}{2})\Gamma(r+\frac{3}{2})} \biggl(\frac{x}{2} \biggr)^{2r+\frac{\nu}{\mathtt{k}}} = \frac{1}{\mathtt{k}} x^{\frac{\nu}{\mathtt{k}}} \mathtt{S}_{\nu-\mathtt {k}, c}^{\mathtt{k}}(x). \end{aligned}$$

This proves (16). We can establish the result (17) by a similar argument as in the proof of (16). We omit the details.

Similarly, from (5), we obtain

20 $$ x\frac{d}{dx}\mathtt{S}_{\nu,c}^{\mathtt{k}}(x)+ \frac{\nu}{\mathtt{k}} \mathtt{S}_{\nu,c}^{\mathtt{k}}(x) =\frac{x}{\mathtt{k}} \mathtt{S}_{{\nu-\mathtt{k}}}^{\mathtt{k}}(x) $$

and

21 $$ x\frac{d}{dx}\mathtt{S}_{\nu,c}^{\mathtt{k}}(x)- \frac{\nu}{\mathtt{k}} \mathtt{S}_{\nu,c}^{\mathtt{k}}(x) =\frac{x {(x/2)}^{\frac{\nu}{\mathtt{k}}}}{ \sqrt{\pi} \Gamma_{\mathtt{k}} (\nu+\frac{\mathtt{3 k}}{2})} -c x\mathtt{S}_{\nu+\mathtt{k}, c}^{\mathtt{k}}(x). $$

Adding and subtracting each side of (20) and (21) yields, respectively, the results (18) and (19). □

## Integral representations

Here, we present two integral representations for the function $\mathtt {S}_{\nu, c}^{\mathtt{k}}$.

### Theorem 3

*Let* $\mathtt{k} \in\mathbb{R}^{+}$, $\Re(\nu )> -\frac{\mathtt{k}}{2}$, *and* $\alpha\in\mathbb{R} \setminus\{0\}$. *Then*

22 $$\begin{aligned} \mathtt{S}_{\nu, \alpha^{2}}^{\mathtt{k}} (x) =\frac{2\sqrt{\mathtt {k}}}{\alpha^{2}\sqrt{\pi}\Gamma_{\mathtt{k}} (\nu+\frac{\mathtt {k}}{2} )} \biggl(\frac{x }{2} \biggr)^{\frac{\nu}{\mathtt{k}}} \int _{0}^{1} \bigl(1-t^{2} \bigr)^{\frac{\nu}{\mathtt{k}}-\frac{1}{2}} \sin \biggl(\frac {\alpha x t}{ \sqrt{\mathtt{k}}} \biggr) \,dt \end{aligned}$$

*and*

23 $$\begin{aligned} \mathtt{S}_{\nu, -\alpha^{2}}^{\mathtt{k}} (x) =\frac{2\sqrt{\mathtt {k}}}{\sqrt{\pi}\Gamma_{\mathtt{k}} (\nu+\frac{\mathtt{k}}{2} )} \biggl(\frac{x }{2} \biggr)^{\frac{\nu}{\mathtt{k}}} \int_{0}^{1} \bigl(1-t^{2} \bigr)^{\frac{\nu}{\mathtt{k}}-\frac{1}{2}} \sinh \biggl(\frac{\alpha x t}{ \sqrt{\mathtt{k}}} \biggr) \,dt. \end{aligned}$$

*In particular*, *we have*

24 $$ 1-\cos \biggl(\frac{\alpha x}{\sqrt{\mathtt{k}}} \biggr)= \frac{\alpha }{\mathtt{k}}\sqrt{ \frac{\pi x}{2}}\mathtt{S}_{\frac{1}{2}, \alpha ^{2}}^{\mathtt{k}} (x) $$

*and*

25 $$ \cosh \biggl(\frac{\alpha x}{\sqrt{\mathtt{k}}} \biggr)-1= \frac{\alpha }{\mathtt{k}} \sqrt{\frac{\pi x}{2}}\mathtt{S}_{\frac{\nu}{\mathtt{k}}, -\alpha^{2}}^{\mathtt{k}} (x). $$

### Proof

We begin by recalling the k-beta function (see [1])

26 $$\begin{aligned} &\mathtt{B}_{\mathtt{k}}(x, y)=\frac{ \Gamma_{\mathtt{k}}(x) \Gamma _{\mathtt{k}}(y)}{\Gamma_{\mathtt{k}}(x+y)}= \frac{1}{\mathtt{k}} \int _{0}^{1} t^{\frac{x}{\mathtt{k}}-1}(1-t)^{\frac{y}{\mathtt{k}}-1} \,dt \\ &\quad \bigl(\mathtt{k} \in\mathbb{R}^{+}; \min\bigl\{ \Re(x), \Re(y)\bigr\} >0 \bigr). \end{aligned}$$

Replacing *t* by $t^{2}$ on the right-hand-sided integral in (26), we obtain

27 $$\begin{aligned} \mathtt{B}_{\mathtt{k}}(x, y)=\frac{2}{\mathtt{k}} \int_{0}^{1} t^{\frac {2x}{\mathtt{k}}-1}\bigl(1-t^{2} \bigr)^{\frac{y}{\mathtt{k}}-1} \,dt. \end{aligned}$$

Setting $x= (r+1)\mathtt{k}$ and $y= \nu+\mathtt{k}/2$ in (27) gives

28 $$\begin{aligned} \frac{ 1}{\Gamma_{\mathtt{k}}(r \mathtt{k}+\nu+\mathtt{k})}=\frac {2}{\Gamma_{\mathtt{k}} ( (r+1 ) \mathtt{k} ) \Gamma _{\mathtt{k}} (\nu+\frac{\mathtt{k}}{2} ) } \int_{0}^{1} t^{2r}\bigl(1-t^{2} \bigr)^{\frac{\nu}{\mathtt{k}}-\frac{1}{2}} \,dt. \end{aligned}$$

Applying the known identity

29 $$\begin{aligned} \Gamma_{\mathtt{k}}(\mathtt{k} x)= \mathtt{k}^{x-1} \Gamma(x) \end{aligned}$$

and the Legendre duplication formula (see [4, 7, 9])

30 $$ \Gamma {(z)}\Gamma { \bigl(z+\tfrac{1}{2} \bigr)}= 2^{1-2z} \sqrt{\pi} \Gamma {(2z)} $$

to the function $\mathtt{S}_{\nu, c}^{\mathtt{k}}$ with (28), we get

31 $$ \mathtt{S}_{\nu, c}^{\mathtt{k}} (x) = \frac{2\sqrt{\mathtt{k}}}{\sqrt{\pi}\Gamma_{\mathtt{k}} (\nu +\frac{\mathtt{k}}{2} )} \biggl(\frac{x }{2} \biggr)^{\frac{\nu }{\mathtt{k}}} \int_{0}^{1} \bigl(1-t^{2} \bigr)^{\frac{\nu}{\mathtt{k}}-\frac{1}{2}} \sum_{r=0}^{\infty}\frac{(-c)^{r} }{ (2r+1)!} \biggl(\frac{x t}{ \sqrt{\mathtt {k}}} \biggr)^{2r+1} \,dt. $$

Finally, setting $c=\pm\alpha^{2}$ $(\alpha\in\mathbb{R}\setminus\{0\} )$ in (31) yields, respectively, the desired results (22) and (23). Further, setting $\nu=\mathtt{k}/2$ in (22) and (23) yields, respectively, the desired results (24) and (25). □

## Results and discussion

We introduce a k-Struve function and investigate its various properties, including mainly certain inequalities associated with this function. One of the inequalities given here is pointed out to be related to the so-called classical Turán-type inequality, whose many variants have been investigated. We also present a differential equation, several recurrence relations, and integral representations for this k-Struve function.

## Conclusions

The results presented here are sure to be new and potentially useful. Since the research subject here and its related ones are competitive, the content of this paper may attract interested readers who have been interested in this and related research subjects.
